# Supplementary material for: Rare events in generalized Lévy Walks and the Big Jump principle
Source: Sci Rep. 2020 Feb 17;10:2732. doi: 10.1038/s41598-020-59187-w (PMC7026067; doi:10.1038/s41598-020-59187-w)
Supplement: Supplementary file 1 — Supplementary Information. [file 41598_2020_59187_MOESM1_ESM.pdf]

Rare events in generalized Lévy Walks and the Big Jump principle.
SUPPLEMENTARY MATERIALS

Alessandro Vezzani,[1,2] Eli Barkai,[3] and Raffaella Burioni[2,4]
[1]IMEM, CNR Parco Area delle Scienze 37/A 43124 Parma
[2]Dipartimento di Matematica, Fisica e Informatica Università degli Studi di Parma, viale G.P.Usberti 7/A, 43100 Parma, Italy
[3]Department of Physics, Institute of Nanotechnology and Advanced Materials, Bar-Ilan University, Ramat-Gan, 52900, Israel
[4]INFN, Gruppo Collegato di Parma, viale G.P. Usberti 7/A, 43100 Parma, Italy

I. SHORT DISTANCE SCALING

We derive here the bulk behavior of the PDF $P(R,T)$ for the generalized Lévy Walk model, showing the non trivial dependence on the exponents α and ν described in Equations (6-7) of the main text.

Let us call $Q(R,T)$ the probability of making a jump at position R and time T. One can write:

$$Q(R,T) = \delta(R)\delta(T) + \frac{1}{2}\int \left[Q(R-ct^\nu, T-t) + Q(R+ct^\nu, T-t)\right]\lambda(t)dt \tag{a}$$

The probability $P(R,T)$ can be reconstructed from $Q(R,T)$ taking into account that a walker can arrive in R only with a step of duration $t_2 > t_1$, with $T - t_1$ being the time when the jump of duration t_2 has been drawn from $\lambda(t_2)$. Then:

$$P(R,T) = \int dt_1 \frac{1}{2}\left[Q(R-ct_2^{\nu-\eta}t_1^\eta, T-t_1) + Q(R+ct_2^{\nu-\eta}t_1^\eta, T-t_1)\right]\int_{t_1}^\infty dt_2\lambda(t_2) \tag{b}$$

Let us consider $\tilde{Q}(k,s)$, i.e. the Fourier transform with respect R and Laplace transform with respect T of $Q(R,T)$. From Eq. (a):

$$\tilde{Q}(k,s) = \frac{1}{1-\tilde{\lambda}(k,s)} \tag{c}$$

where $\tilde{\lambda}(k,s)$ is:

$$\tilde{\lambda}(k,s) = \int dt \frac{\lambda(t)}{2}\left(e^{-st+ikct^\nu} + e^{-st-ikct^\nu}\right). \tag{d}$$

From Eq. (b) we obtain the Laplace Fourier transform of $P(R,T)$

$$\tilde{P}(k,s) = \tilde{Q}(k,s)\tilde{\gamma}(k,s) \tag{e}$$

where

$$\tilde{\gamma}(k,s) = \int_0^\infty dt_1 e^{-st_1}\int_{t_1}^\infty dt_2 \frac{\lambda(t')}{2}\left(e^{ikct_2^{\nu-\eta}t_1^\eta} + e^{-ikct_2^{\nu-\eta}t_1^\eta}\right). \tag{f}$$

Now we can expand $\tilde{\lambda}(k,s)$ and $\tilde{\gamma}(k,s)$ for small s and k. Keeping only the leading terms in Eq. (c) and (c) for $\alpha > 2\nu$ and $\alpha > 1$ gives:

$$\tilde{P}(k,s) = \frac{\langle t\rangle}{s\langle t\rangle + (1/2)k^2\langle t^{2\nu}\rangle}. \tag{g}$$

and $P(R,T)$ is a Gaussian centered in the origin

$$P(R,T) = \sqrt{\frac{\langle t\rangle}{2\pi Tc^2\langle t^{2\nu}\rangle}}e^{-\frac{R^2\langle t\rangle}{2Tc^2\langle t^{2\nu}\rangle}} = \frac{1}{T^{1/2}}G\left(\frac{R}{T^{1/2}}\right). \tag{h}$$

where $G(\cdot)$ is a Gaussian scaling function and the characteristic length of the process $\ell(T)$ grows as $\ell(T) \sim T^{1/2}$. If $2\nu > \alpha$ and $\alpha > 1$ expanding $\tilde{\lambda}(k,s)$ and $\tilde{\gamma}(k,s)$ we obtain:

$$\tilde{P}(k,s) = \frac{\langle t \rangle}{s\langle t \rangle + A_\alpha |k|^{\alpha/\nu}}. \tag{i}$$

where $A_\alpha = \int_0^\infty (1-\cos(t^\nu)) \frac{\tau_0^\alpha}{t^{1+\alpha}} dt$ is a constant. The inverse Fourier-Laplace transform gives:

$$P(R,T) = \frac{1}{T^{\nu/\alpha}} L_{\nu/\alpha}\left(\frac{R}{T^{\nu/\alpha}}\right). \tag{j}$$

where $L_{\nu/\alpha}(\cdot)$ is a Lévy stable scaling function and the characteristic length now is $\ell(t) \sim t^{\nu/\alpha}$. For $2\nu < \alpha$ and $\alpha < 1$ we obtain:

$$\tilde{P}(k,s) = \frac{s^{\alpha-1} B_\alpha}{s^\alpha B_\alpha + (1/2) k^2 \langle t^{2\nu} \rangle}. \tag{k}$$

with $B_\alpha = \int_0^\infty (1-e^{-t}) \frac{\tau_0^\alpha}{t^{1+\alpha}} dt$. So the PDF scales as:

$$P(R,T) = \frac{1}{T^{\alpha/2}} C_\alpha \left(\frac{R}{T^{\alpha/2}}\right). \tag{l}$$

where $C_\alpha(\cdot)$ for $\alpha < 1$ is the scaling function of CTRW with infinite average waiting times. Finally for $\alpha < 2\nu$ and $\alpha < 1$ $\tilde{\lambda}(k,s)$ and $\tilde{\gamma}(k,s)$ cannot be expanded to first order neither in k nor in s. In this case:

$$P(R,T) = \frac{1}{T^\nu} f^* \left(\frac{R}{cT^\nu}\right). \tag{m}$$

where $f^*(\cdot)$ is a non universal scaling function depending on α, ν, and μ.

II. THE BIG JUMP IN GENERALIZED LÉVY WALKS

Here, we perform the explicit calculation of the tail of the distribution $B(R,T)$ applying the big jump principle to the generalized Lévy Walk model. The process has been described in Figure 1 of the main text.

Let us first consider the case $\alpha > 1$ and $\nu > 1/2$ where both processes in Figure 1 are relevant. In particular, $\mathcal{P}(R|T,L,T_w)$ can be written introducing the Kronecker δ-function and the Heaviside θ-function:

$$\mathcal{P}(R|T,t,T_w) = \delta(R - ct^{\nu-\eta}(T-T_w)^\eta)\theta(t-(T-T_w)) + \delta(R-ct^\nu)\theta((T-T_w)-t) \tag{n}$$

where the first and the second terms correspond to the paths in panels (**a**) and (**b**) of Figure 1 respectively. Since $\alpha > 1$, the jump rate is constant ($n_R(T_w) = \langle t \rangle^{-1}$) and $p_{\text{tot}}(t,T_w) = \lambda(t)/\langle t \rangle$. Plugging Eq. (n) into formula (3) we obtain:

$$\begin{aligned} B(R,T) &= B_0(R,T) + B_1(R,T) \\ B_0(R,T) &= \int_0^T \frac{dT_w}{\langle t \rangle} \int_0^\infty \frac{dt \tau_0^\alpha}{t^{1+\alpha}} \delta(R - ct^{\nu-\eta}(T-T_w)^\eta)\theta(t-(T-T_w)), \\ B_1(R,T) &= \int_0^T \frac{dT_w}{\langle t \rangle} \int_0^\infty \frac{dt \tau_0^\alpha}{t^{1+\alpha}} \delta(R-ct^\nu)\theta((T-T_w)-t) \end{aligned} \tag{o}$$

so that $B_0(R,T)$ and $B_1(R,T)$ are generated by the first and the second process illustrated in Figure 1 (main) respectively. Defining $y = ct^\nu$ in $B_1(R,T)$ we obtain:

$$\begin{aligned} B_1(R,T) &= \int_0^T \frac{dT_w}{\langle t \rangle} \int_0^\infty \frac{dy c^{\alpha/\nu} \tau_0^\alpha}{\nu y^{1+\alpha/\nu}} \delta(R-y)\theta((T-T_w)-(y/c)^{1/\nu}) = \int_0^T \frac{dT_w c^{\alpha/\nu} \tau_0^\alpha}{\nu \langle t \rangle R^{1+\alpha/\nu}} \theta((T-T_w)-(R/c)^{1/\nu}) \\ &= \begin{cases} \frac{c^{\alpha/\nu}\tau_0^\alpha \left(T - \left(\frac{R}{c}\right)^{1/\nu}\right)}{\nu \langle t \rangle R^{1+\alpha/\nu}} = \frac{\tau_0^\alpha \left(1 - \left(\frac{R}{cT^\nu}\right)^{1/\nu}\right)}{T^{\alpha-1+\nu} c\nu \langle t \rangle \left(\frac{R}{cT^\nu}\right)^{1+\alpha/\nu}} & \text{if } (R/c)^{1/\nu} < T \\ 0 & \text{if } (R/c)^{1/\nu} > T \end{cases} \end{aligned} \tag{p}$$

In $B_0(R,T)$, we replace the integration variable T_w with $T_2 = T - T_w$ and then t with $y = ct^{\nu-\eta}T_2^{\eta}$, for $\eta < \nu$ we obtain:

$$\begin{aligned}
B_0(R,T) &= \int_0^T \frac{dT_2}{\langle t\rangle}\int_{T_2}^{\infty}\frac{dt\tau_0^{\alpha}}{t^{1+\alpha}}\delta(R-ct^{\nu-\eta}T_2^{\eta}) = \int_0^T \frac{dT_2}{\langle t\rangle}\int_{cT_2^{\nu}}^{\infty}\frac{dyT_2^{\frac{\alpha\eta}{\nu-\eta}}c^{\frac{\alpha}{\nu-\eta}}\tau_0^{\alpha}}{(\nu-\eta)y^{1+\frac{\alpha}{\nu-\eta}}}\delta(R-y) \\
&= \int_0^T \frac{dT_2}{\langle t\rangle}\frac{T_2^{\frac{\alpha\eta}{\nu-\eta}}c^{\frac{\alpha}{\nu-\eta}}\tau_0^{\alpha}}{(\nu-\eta)R^{1+\frac{\alpha}{\nu-\eta}}}\theta(R-cT_2^{\nu}) = \int_0^{\min(T,(R/c)^{1/\nu})} dT_2\frac{T_2^{\alpha\eta/(\nu-\eta)}c^{\frac{\alpha}{\nu-\eta}}\tau_0^{\alpha}}{\langle t\rangle(\nu-\eta)R^{1+\frac{\alpha}{\nu-\eta}}} \\
&= \begin{cases} \frac{c^{\alpha/\nu}\tau_0^{\alpha}\left(\frac{R}{c}\right)^{1/\nu}}{(\nu+(\alpha-1)\eta)\langle t\rangle R^{1+\alpha/\nu}} = \frac{\tau_0^{\alpha}\left(\frac{R}{cT^{\nu}}\right)^{1/\nu}}{T^{\alpha-1+\nu}c(\nu+(\alpha-1)\eta)\langle t\rangle\left(\frac{R}{cT^{\nu}}\right)^{1+\alpha/\nu}} & \text{if } (R/c)^{1/\nu} < T \\ \frac{T^{\frac{\alpha\eta}{\nu-\eta}+1}c^{\frac{\alpha}{\nu-\eta}}\tau_0^{\alpha}}{\langle t\rangle(\nu+(\alpha-1)\eta)R^{1+\frac{\alpha}{\nu-\eta}}} = \frac{\tau_0^{\alpha}}{T^{\alpha-1+\nu}c(\nu+(\alpha-1)\eta)\langle t\rangle\left(\frac{R}{cT^{\nu}}\right)^{1+\frac{\alpha}{\nu-\eta}}} & \text{if } (R/c)^{1/\nu} > T \end{cases}
\end{aligned} \tag{q}$$

For $\eta > \nu$ we have:

$$\begin{aligned}
B_0(R,T) &= \int_0^T \frac{dT_2}{\langle t\rangle}\int_{T_2}^{\infty}\frac{dt\tau_0^{\alpha}}{t^{1+\alpha}}\delta(R-ct^{\nu-\eta}T_2^{\eta}) = \int_0^T \frac{dT_2}{\langle t\rangle}\int_0^{cT_2^{\nu}}\frac{dyT_2^{\frac{\alpha\eta}{\nu-\eta}}c^{\frac{\alpha}{\nu-\eta}}\tau_0^{\alpha}}{(\eta-\nu)y^{1+\frac{\alpha}{\nu-\eta}}}\delta(R-y) \\
&= \int_0^T \frac{dT_2}{\langle t\rangle}\frac{T_2^{\frac{\alpha\eta}{\nu-\eta}}c^{\frac{\alpha}{\nu-\eta}}\tau_0^{\alpha}}{(\eta-\nu)R^{1+\frac{\alpha}{\nu-\eta}}}\theta(cT_2^{\nu}-R) = \int_{(R/c)^{1/\nu}}^{T} dT_2\frac{T_2^{\frac{\alpha\eta}{\nu-\eta}}c^{\frac{\alpha}{\nu-\eta}}\tau_0^{\alpha}}{\langle t\rangle(\eta-\nu)R^{1+\frac{\alpha}{\nu-\eta}}} \\
&= \begin{cases} \frac{\tau_0^{\alpha}}{T^{\alpha-1+\nu}c(\nu+(\alpha-1)\eta)\langle t\rangle}\left(\frac{\left(\frac{R}{ct^{\nu}}\right)^{1/\nu}}{\left(\frac{R}{ct^{\nu}}\right)^{1+\alpha/\nu}} - \frac{1}{\left(\frac{R}{ct^{\nu}}\right)^{1+\frac{\alpha}{\nu-\eta}}}\right) & \text{if } (R/c)^{1/\nu} < T \\ 0 & \text{if } (R/c)^{1/\nu} > T \end{cases}
\end{aligned} \tag{r}$$

Finally for $\nu = \eta$:

$$\begin{aligned}
B_0(R,T) &= \int_0^T \frac{dT_2}{\langle t\rangle}\int_{T_2}^{\infty}\frac{dt\tau_0^{\alpha}}{t^{1+\alpha}}\delta(R-cT_2^{\nu}) = \int_0^T \frac{dT_2}{\langle t\rangle}\frac{\tau_0^{\alpha}}{\alpha T_2^{\alpha}}\delta(R-cT_2^{\nu}) \\
&= \int_0^{cT^{\nu}} \frac{dy\left(\frac{y}{c}\right)^{1/\nu-1}\tau_0^{\alpha}}{c\alpha\langle t\rangle\alpha\nu\left(\frac{y}{c}\right)^{\alpha/\nu}}\delta(R-y) \\
&= \begin{cases} \frac{\tau_0^{\alpha}\left(\frac{R}{c}\right)^{1/\nu}}{\nu\alpha c\langle t\rangle\left(\frac{R}{c}\right)^{1+\alpha/\nu}} = \frac{\tau_0^{\alpha}\left(\frac{R}{cT^{\nu}}\right)^{1/\nu}}{T^{\alpha-1+\nu}c\nu\alpha\langle t\rangle\left(\frac{R}{cT^{\nu}}\right)^{1+\alpha/\nu}} & \text{if } (R/c)^{1/\nu} < T \\ 0 & \text{if } (R/c)^{1/\nu} > T \end{cases}
\end{aligned} \tag{s}$$

Eq.s (o-s) define the function $F(x)$ in Eq. (8). In particular, the non universal scaling function $F(x)$ depends on the exponents α, ν and η and it is non-analytic at $x = 1$. For $\eta \neq \nu$ $F(x)$ is continuous but non derivable at $x = 1$. In particular, if $\eta > \nu$, $F(x) > 0$ for all x. When $\eta < \nu$, $F(x) > 0$ for $x < 1$ and $F(x) = 0$ for $x > 1$. Finally, for $\eta = \nu$, $F(x)$ is discontinuous, dropping to 0 at $x = 1$ with $\lim_{x\to 1^-} F(x) > 0$ and $F(x) = 0$ for $x > 1$.

For $\alpha > 1$, $\nu < 1/2$ and $\eta < \nu$ the jump rate is again constant and $p_{\rm tot}(t, T_w) = \lambda(t)/\langle t\rangle$; however only the first process in Figure 1 (main) provide a contribution. In this case $\mathcal{P}(R, T, t, T_w) = \delta(R - ct^{\nu-\eta}(T - T_w)^{\eta})\theta(t - (T - T_w))$ and we get the same result of Eq. (q) (for $R > cT^{\nu}$), i.e. Eq. (9).

For $\alpha < 1$ we have $p_{\rm tot}(t, T_w) = C_\alpha T_w^{\alpha-1}/t^{\alpha+1}$. Taking into account only of the first process in Figure 1 (main) for $\eta < \nu$ we have:

$$\begin{aligned}
B(R,T) &= \int_0^T dT_w \int dt p_{\rm tot}(t,T_w)\delta(R-ct^{\nu-\eta}(T-T_w)^{\eta})\theta(t-(T-T_w)) \\
&= \int_0^T dT_2 C_\alpha(T-T_2)^{\alpha-1}\int_{T_2}^{\infty}\frac{dt}{t^{1+\alpha}}\delta(R-ct^{\nu-\eta}T_2^{\eta}) \\
&= \int_0^T \frac{dT_2 C_\alpha(T-T_2)^{\alpha-1}}{\nu-\eta}\int_{cT_2^{\nu}}^{\infty}\frac{dyT_2^{\frac{\alpha\eta}{\nu-\eta}}c^{\frac{\alpha}{\nu-\eta}}}{y^{1+\frac{\alpha}{\nu-\eta}}}\delta(R-y) = \int_0^T \frac{dT_2 C_\alpha(T-T_2)^{\alpha-1}T_2^{\frac{\alpha\eta}{\nu-\eta}}c^{\frac{\alpha}{\nu-\eta}}}{(\nu-\eta)R^{1+\frac{\alpha}{\nu-\eta}}} \\
&= \frac{T^{\frac{\nu\alpha}{\nu-\eta}}c^{\frac{\alpha}{\nu-\eta}}C_\alpha\int_0^1 dx(1-x)^{\alpha-1}x^{\frac{\alpha\eta}{\nu-\eta}}}{(\nu-\eta)R^{1+\frac{\alpha}{\nu-\eta}}}
\end{aligned} \tag{t}$$

where $T_2 = T - T_w$, $y = ct^{\nu-\eta}T_2^{\eta}$ $x = T_2/T$, and we use the fact that $R \gg \ell(T) \gtrsim cT^{\nu} > cT_2^{\nu}$ to fix $\delta(R-y)$. Setting $D_\alpha = C_\alpha\int_0^1 dx(1-x)^{\alpha-1}x^{\frac{\alpha\eta}{\nu-\eta}}$ we obtain Eq. (10).
